# Longitudinal evolution of motor and non-motor symptoms in early-stage multiple system atrophy: a 2-year prospective cohort study

**DOI:** 10.1186/s12916-022-02645-1

**Published:** 2022-11-17

**Authors:** Lingyu Zhang, Yanbing Hou, Bei Cao, Qianqian Wei, Ruwei Ou, Kuncheng Liu, Junyu Lin, Tianmi Yang, Yi Xiao, Yongping Chen, Wei Song, Bi Zhao, Huifang Shang

**Affiliations:** Department of Neurology, Laboratory of Neurodegenerative Disorders, Rare Diseases Center, West China Hospital, Sichuan University, Chengdu, 610041 Sichuan China

**Keywords:** Multiple system atrophy, Motor symptoms, Non-motor symptoms, Prospective study

## Abstract

**Background:**

The progression of motor and non-motor symptoms (NMS) and the sensitivity of each item of the Unified Multiple System Atrophy Rating Scale (UMSARS) to change remain unclear in Chinese patients with early-stage multiple system atrophy (MSA). We investigated the evolution of motor symptoms and NMS in early-stage MSA and the sensitivity of each item included in the UMSARS to change over a 2-year follow-up.

**Methods:**

Motor symptoms and NMS were recorded at baseline and at 1- and 2-year follow-ups based on the UMSARS and the NMS scale. Generalized estimating equation models were used. The sensitivity of an item included in the UMSARS to change was assessed by calculating a standardized effect using the mean annual change divided by the standard deviation of the change.

**Results:**

We enrolled 246 consecutive patients with MSA and 97 MSA completed the 2-year follow-up. The mean total UMSARS score increased by 11.90 and 22.54 points at the 1- and 2-year follow-ups, respectively. UMSARS-I items associated with motor functions were more sensitive to change and those associated with autonomic dysfunction showed less sensitivity to change. Items 4 (tremor at rest), 5 (action tremor), and 3 (ocular motor dysfunction) of the UMSARS-II were less sensitive to change. The prevalence and severity of NMS significantly increased over the 2-year follow-up.

**Conclusions:**

We observed significant progression in motor symptoms and NMS in patients with early-stage MSA. Our results provide useful information to support the revision of the UMSARS.

**Supplementary Information:**

The online version contains supplementary material available at 10.1186/s12916-022-02645-1.

## Background

Multiple system atrophy (MSA) is a rare, adult-onset, progressive neurodegenerative disorder; MSA with predominantly parkinsonian features is designated as MSA-P and that with predominant features of cerebellar ataxia as MSA-C [1]. The etiology of this condition remains unclear, and treatment is ineffective in most cases. MSA progresses rapidly and is associated with poor prognosis, with a median survival time of 6–10 years [2, 3].

An increasing number of studies have reported the progression of motor symptoms in MSA based on the Unified Multiple System Atrophy Rating Scale (UMSARS) [4], which was developed nearly 20 years ago for the assessment of disease severity in patients with MSA [5, 6]. However, a few studies have observed that some items included in the UMSARS are not sensitive to MSA progression [7–9]. The UMSARS consists of four parts referred to as UMSARS-I (a historical review of both motor and autonomic disabilities), UMSARS-II (motor examination), UMSARS-III (autonomic examination), and UMSARS-IV (global disability) [4]. UMSARS-I and II are commonly used as primary endpoints in clinical trials [10]. Therefore, longitudinal observation of changes in each item included in the UMSARS-I and II is clinically significant, particularly during the early stages of the disease.

In addition to motor symptoms, patients with MSA frequently present with non-motor symptoms (NMS), which negatively affect patients’ quality of life [11, 12]. Most studies have investigated the clinical characteristics of NMS in patients with MSA, who show a high prevalence of depression, anxiety, sleep problems, cognitive impairment, and fatigue, among other such features [11, 13–15]. However, most of the aforementioned studies were cross-sectional in design. A recent small-scale Spanish study reported significant progression in urinary and sexual dysfunction, as well as sleep difficulties in patients with MSA, who underwent a 2-year follow-up [14]. NMS progression in patients with early-stage MSA remains largely unknown.

To date, the progression of motor symptoms and NMS and the sensitivity of each item included in the UMSARS to change remain unclear in Chinese patients with early-stage MSA, and a better understanding of the motor symptom and overall NMS progression in early-stage MSA may be useful for clinical management and for clinical trials. Therefore, we performed a 2-year prospective cohort study to investigate the progression of motor symptoms and overall NMS, as well as the sensitivity of each item of the UMSARS to change in patients with early-stage MSA.

## Methods

### Study design and population

In this prospective cohort study, we recruited consecutive patients with MSA from the Department of Neurology, West China Hospital of Sichuan University, between January 2014 and May 2021. All patients had a baseline disease duration of <3 years and underwent regular face-to-face follow-ups every year, with the final follow-up in April 2022. Upon completion of follow-up, patients who met the criteria for a probable diagnosis of MSA [16], in whom follow-up data for each year were available, were included in the present study. To exclude common forms of spinocerebellar ataxia (SCA), we performed screening for *SCA* genes including *SCA1*, *SCA2*, *SCA3*, *SCA6*, and *SCA7*. All patients underwent brain magnetic resonance imaging to exclude other neurological disorders.

**Fig. 1:**
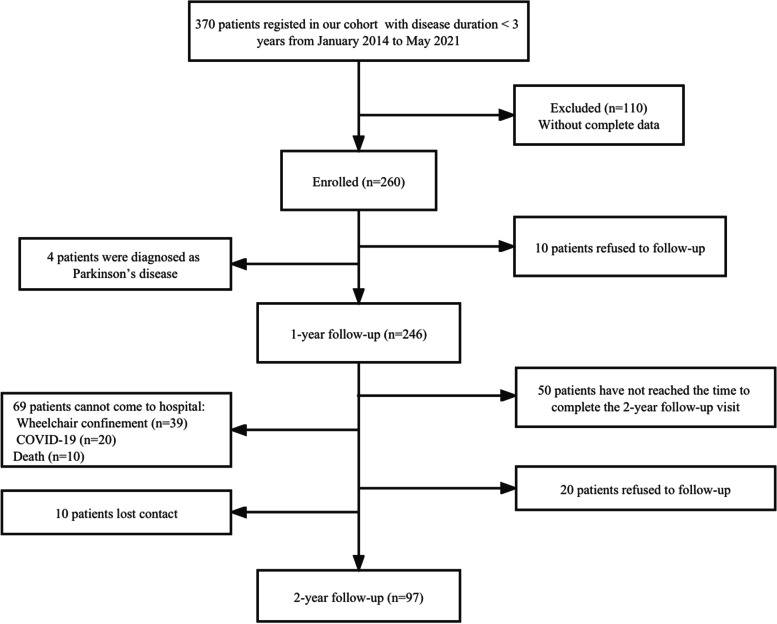
Study flow diagram

Patients underwent face-to-face interviews by neurologists at baseline and at 1- and 2-year follow-ups. The number of patients included at baseline was 260; 10 patients who refused to follow up and four patients diagnosed with Parkinson’s disease during the 1-year follow-up were excluded from the study. Therefore, only 246 patients completed the 1-year follow-up evaluation. Furthermore, 50 patients had not reached the time to complete the 2-year follow-up; 69 patients could not visit the hospital owing to wheelchair confinement, COVID-19, or death (mortality rate: 4.1%); 20 patients refused to follow up; and 10 patients were lost to follow-up. Therefore, eventually, 97 patients completed the 2-year follow-up (Fig. 1).

### Evaluation protocol

Baseline clinical information, including age, sex, age of onset, treatment, and disease duration, was recorded. The total levodopa equivalent daily dose (LEDD) was calculated based on details reported by a previous study [17]. Disease duration was defined as the time between disease onset and evaluation. Disease onset was defined as the initial presentation of any motor symptoms (parkinsonism or cerebellar dysfunction) or autonomic features, except erectile dysfunction.

Patients with MSA underwent evaluation for disease severity and NMS at baseline and at 1- and 2-year follow-ups. Disease severity was evaluated using the UMSARS, and the total UMSARS score was calculated as the sum of parts I and II. NMS was assessed using the Non-Motor Symptoms Scale (NMSS) as previously reported [11]. The NMSS contains 30 items classified into the following nine domains: cardiovascular, sleep/fatigue, mood/apathy, perceptual problems/hallucinations, attention/memory, gastrointestinal, urinary, sexual function, and miscellaneous. Each item was scored as a multiple of severity (0–3) and frequency (1–4), and the maximum NMS score was 360. The prevalence of each domain of the NMSS was calculated using the percentage of patients with scores ≥1 in each NMSS domain.

### Statistical analysis

All continuous data are presented as mean and standard deviation (SD), or median and interquartile range, and categorical variables as counts (percentages). Student’s *t*-test or chi-squared test was used to compare the variables between patients with MSA who dropped out at follow-up and those who did not. A generalized estimating equation model with an exchangeable working correlation structure was used to investigate significant longitudinal motor symptoms and NMS changes in MSA after adjusting by baseline age and sex, in this longitudinal observational study [14]. The Bonferroni correction was used for multiple comparisons. The sensitivity of an item included in the UMSARS-I and II or of a domain of the NMSS to change was assessed by calculating a standardized effect (SE) using the mean annual change divided by the SD of the change [9]. The higher the SE score, the greater the sensitivity of the item to change. All data analyses were performed using the IBM SPSS Statistics software (version 26.0). *P* value < 0.05 was considered statistically significant.

## Results

### Baseline demographics

A total of 246 consecutive patients with MSA at baseline completed the 1-year follow-up and only 97 completed the 2-year follow-up in this study. Additional file 1: Table S1 shows patients’ baseline demographic data. Patients’ mean age was 59.41±7.68 years, and the mean disease duration was 1.72±0.76 years. The study included 53.7% (132) male, and 54.5% (134) of patients had MSA-C. There were no significant differences in the clinical demographic characteristics between patients with MSA who dropped out at follow-up and those who did not except the LEDD. And patients who dropped out had higher doses of LEDD than those who did not (195.64±252.98 vs. 96.52±174.28, *P*=0.001).

### Unified Multiple System Atrophy Rating Scale progression

**Fig. 2:**
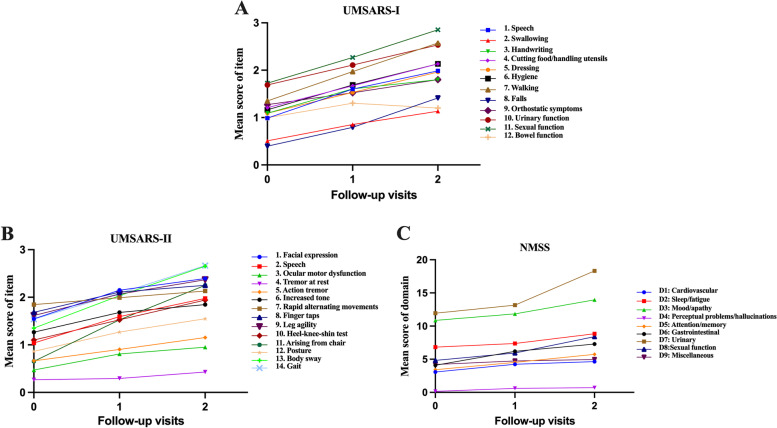
**A** Progression of items included in the UMSARS-I over the 2-year follow-up. **B** Progression of items included in the UMSARS-II over the 2-year follow-up. **C** Progression of domains included in the NMSS over the 2-year follow-up. UMSARS, Unified Multiple System Atrophy Rating Scale; NMSS, Non-Motor Symptoms Scale

The total UMSARS, the UMSARS-I and II scores, and scores for each item of the UMSARS progressed significantly during the longitudinal follow-up, except for the UMSARS-II item 4 (tremor at rest) (Table 1, Fig. 2A, B). Post hoc tests showed that these items increased from baseline until 1- and 2-year follow-ups. The mean total UMSARS score increased by 11.90 and 22.54 points at the 1- and 2-year follow-ups.

**Table 1:** The comparison of UMSARS items at baseline and 1- and 2-year follow-ups

UMSARS item	Baseline (*n*=246)		1-year follow-up (*n*=246)		2-year follow-up (*n*=97)		*p* value	Post hoc tests
	Median	IQR	Median	IQR	Median	IQR		
Part I								
1. Speech	1	0	1	1	2	2	<0.001^a^	**a,b,c**
2. Swallowing	0	1	1	1	1	1	<0.001^a^	**a,b,c**
3. Handwriting	1	0	1	1	2	2	<0.001^a^	**a,b,c**
4. Cutting food/handling utensils	1	1	2	1	2	2	<0.001^a^	**a,b,c**
5. Dressing	1	0	1	1	2	2	<0.001^a^	**a,b,c**
6. Hygiene	1	1	2	1	2	2	<0.001^a^	**a,b,c**
7. Walking	1	1	2	2	3	1	<0.001^a^	**a,b,c**
8. Falls	0	1	0	1	1	2	<0.001^a^	**a,b,c**
9. Orthostatic symptoms	1	2	1	3	2	2	<0.001^a^	**a,b**
10. Urinary function	2	2	3	2	3	1	<0.001^a^	**a,b,c**
11. Sexual function	1	4	3	4	4	1	<0.001^a^	**a,b,c**
12. Bowel function	1	2	1	2	1	2	<0.001^a^	**a,b**
Part II								
1. Facial expression	2	1	2	0	2	1	<0.001^a^	**a,b,c**
2. Speech	1	0	1	1	2	2	<0.001^a^	**a,b,c**
3. Ocular motor dysfunction	0	1	1	1	1	2	<0.001^a^	**a,b**
4. Tremor at rest	0	0	0	0	0	1	0.158	
5. Action tremor	1	1	1	1	1	1	<0.001^a^	**a,b,c**
6. Increased tone	1.5	2	2	1	2	1	<0.001^a^	**a,b**
7. Rapid alternating movements	1	1	2	0	2	1	<0.001^a^	**a,b,c**
8. Finger taps	2	1	2	1	2	1	<0.001^a^	**a,b,c**
9. Leg agility	2	1	2	0	2	1	<0.001^a^	**a,b,c**
10. Heel-knee-shin test	1	2	2	1	2	1	<0.001^a^	**a,b,c**
11. Arising from chair	0	1	1	1	2	3	<0.001^a^	**a,b,c**
12. Posture	1	1	1	1	1	1	<0.001^a^	**a,b,c**
13. Body sway	1	1	2	2	3	2	<0.001^a^	**a,b,c**
14. Gait	1	1	2	2	3	1	<0.001^a^	**a,b,c**
UMSARS-I	13	7	18	10	24	11	<0.001^a^	**a,b,c**
UMSARS-II	15	8	22	9	26	12	<0.001^a^	**a,b,c**
UMSARS total	28	15	40	18	48	22	<0.001^a^	**a,b,c**

Post hoc tests (Bonferroni correction)

Baseline vs 1-year follow-up: **a**, significant

Baseline vs 2-year follow-up: **b**, significant

1- vs 2-year follow-up: **c**, significant

*UMSARS* Unified Multiple System Atrophy Rating Scale, *IQR* interquartile range

^a^Significant difference

The sensitivity to change of each item of UMSARS in patients with MSA is shown in Table 2. UMSARS-I items associated with key motor functions, including speech (SE, 0.80/1.13), handwriting (SE, 0.56/0.92), cutting food/handling utensils (SE, 0.58/0.93), dressing (SE, 0.61/0.94), hygiene (SE, 0.67/1.01), and walking (SE, 0.73/1.41) were more sensitive to change in contrast to items associated with autonomic dysfunction (orthostatic symptoms (SE, 0.19/0.41), as well as urinary (SE, 0.35/0.79), sexual (SE, 0.26/0.64), and bowel function (SE, 0.32/0.31)) across 1- and 2-year follow-ups. With regard to UMSARS-II evaluation, items such as arising from chair (SE, 0.87/1.36), speech (SE, 0.74/1.07), and gait (SE, 0.69/1.23) were most sensitive to change, in contrast to items including tremor at rest (SE, 0.06/0.18), action tremor (SE, 0.23/0.48), and ocular motor dysfunction (SE, 0.32/0.48), which showed the least sensitivity to change across 1- and 2-year follow-ups.

**Table 2:** The sensitivity to change of each item of UMSARS in patients with MSA

UMSARS item	Baseline to 1-year follow-up (*n*=246)			Baseline to 2-year follow-up (*n*=97)			1- to 2-year follow-up (*n*=97)		
	Mean annual change	SD of the change	Standardized effect	Mean annual change	SD of the change	Standardized effect	Mean annual change	SD of the change	Standardized effect
Part I									
1. Speech	0.56	0.71	0.80	0.52	0.46	1.13	0.61	1.30	0.47
2. Swallowing	0.33	0.80	0.42	0.38	0.46	0.84	0.48	0.92	0.52
3. Handwriting	0.46	0.83	0.56	0.41	0.45	0.92	0.57	0.83	0.69
4. Cutting food/handling utensils	0.42	0.73	0.58	0.44	0.48	0.93	0.51	0.85	0.60
5. Dressing	0.40	0.66	0.61	0.44	0.48	0.94	0.51	0.78	0.66
6. Hygiene	0.48	0.71	0.67	0.51	0.50	1.01	0.55	0.78	0.71
7. Walking	0.57	0.78	0.73	0.63	0.44	1.41	0.77	0.75	1.03
8. Falls	0.37	1.00	0.36	0.38	0.63	0.60	0.47	1.40	0.34
9. Orthostatic symptoms	0.22	1.19	0.19	0.31	0.76	0.41	0.23	1.23	0.19
10. Urinary function	0.41	1.17	0.35	0.49	0.62	0.79	0.73	1.28	0.57
11. Sexual function	0.51	1.97	0.26	0.64	1.00	0.64	1.01	1.88	0.54
12. Bowel function	0.30	0.93	0.32	0.17	0.57	0.31	0.09	1.13	0.08
Part II									
1. Facial expression	0.56	0.90	0.62	0.45	0.55	0.83	0.51	0.93	0.55
2. Speech	0.50	0.68	0.74	0.48	0.45	1.07	0.52	0.78	0.67
3. Ocular motor dysfunction	0.31	0.96	0.32	0.21	0.48	0.44	0.09	1.07	0.08
4. Tremor at rest	0.04	0.63	0.06	0.07	0.41	0.18	0.12	0.75	0.15
5. Action tremor	0.21	0.92	0.23	0.23	0.48	0.48	0.30	0.94	0.32
6. Increased tone	0.40	1.15	0.35	0.39	0.59	0.67	0.45	1.11	0.40
7. Rapid alternating movements	0.40	0.85	0.47	0.32	0.49	0.65	0.30	0.82	0.37
8. Finger taps	0.38	0.70	0.55	0.36	0.46	0.77	0.32	0.85	0.37
9. Leg agility	0.42	0.75	0.56	0.44	0.42	1.05	0.48	0.70	0.68
10. Heel-knee-shin test	0.39	0.95	0.41	0.40	0.56	0.71	0.43	0.98	0.44
11. Arising from chair	0.79	0.91	0.87	0.77	0.57	1.36	0.95	1.00	0.95
12. Posture	0.37	0.75	0.49	0.37	0.46	0.81	0.44	0.83	0.53
13. Body sway	0.62	1.00	0.61	0.61	0.64	0.95	0.66	1.11	0.59
14. Gait	0.50	0.73	0.69	0.56	0.45	1.23	0.66	0.68	0.97

*UMSARS* Unified Multiple System Atrophy Rating Scale, *MSA* multiple system atrophy, *SD* standard deviation

### Non-Motor Symptoms Scale progression

The mean score of each domain of the NMSS significantly increased during the follow-up period, except for the miscellaneous domain (Table 3 and Fig. 2C). Post hoc tests showed that the mean score of these NMSS domains increased from baseline until the 2-year follow-up, whereas the mean score associated with perceptual problems/hallucinations, attention/memory, and cardiovascular, gastrointestinal, and sexual function significantly increased from baseline until the 1-year follow-up. The median total NMSS score increased from 41 to 46 after 1 year and to 65 after 2 years. Most NMSS domains (such as sleep/fatigue (SE, 0.08), mood/apathy (SE, 0.06), urinary (SE, 0.11), and miscellaneous (SE, 0.12)) were less sensitive to change from baseline until the 1-year follow-up. However, some domains including attention/memory (SE, 0.53/0.47), gastrointestinal (SE, 0.59/0.38), urinary (SE, 0.83/0.78), and sexual functions (SE, 0.56/0.44) showed greater sensitivity to change from baseline until the 2-year follow-up and 1- to 2-year follow-up (Table 4). Additionally, the frequency of each NMSS domain was significantly increased from baseline until the 1- and 2-year follow-ups (Additional file 1: Table S2).

**Table 3:** The comparison of the NMSS domain at baseline and 1- and 2-year follow-ups

	Baseline (*n*=246)		1-year follow-up (*n*=246)		2-year follow-up (*n*=97)		*p* value	Post hoc tests
NMSS	Median	IQR	Median	IQR	Median	IQR		
D1: Cardiovascular	2	4	2	5	3	7	<0.001^a^	**a,b**
D2: Sleep/fatigue	5	8	6	7	6	9	0.004^a^	**b**
D3: Mood/apathy	4	16	6	14	6	20	0.024^a^	**b**
D4: Perceptual problems/hallucinations	0	0	0	0	0	0	<0.001^a^	**a,b**
D5: Attention/memory	2	5	3	5	4	6	<0.001^a^	**a,b,c**
D6: Gastrointestinal	2.5	6	4	7	5	8	<0.001^a^	**a,b,c**
D7: Urinary	9	18	9.5	16	17	18	<0.001^a^	**b,c**
D8: Sexual function	0	8	2	8	8	14	<0.001^a^	**a,b,c**
D9: Miscellaneous	2	8	4	7	3	6	0.053	
NMSS total score	41	45	46	47	65	50	<0.001^a^	**a,b,c**

Post hoc tests (Bonferroni correction)

Baseline vs 1-year follow-up: **a**, significant

Baseline vs 2-year follow-up: **b**, significant

1- vs 2-year follow-up: **c**, significant

*NMSS* Non-Motor Symptoms Scale, *IQR* interquartile range

^a^Significant difference

**Table 4:** The sensitivity to change of each domain of NMSS in patients with MSA

NMSS	Baseline to 1-year follow-up (*n*=246)			Baseline to 2-year follow-up (*n*=97)			1- to 2-year follow-up (*n*=97)		
	Mean annual change	SD of the change	Standardized effect	Mean annual change	SD of the change	Standardized effect	Mean annual change	SD of the change	Standardized effect
D1: Cardiovascular	1.09	3.84	0.28	0.95	2.42	0.39	0.37	4.03	0.09
D2: Sleep/fatigue	0.52	6.66	0.08	1.08	3.55	0.30	1.87	6.91	0.27
D3: Mood/apathy	0.93	14.85	0.06	2.08	7.37	0.28	3.73	14.36	0.26
D4: Perceptual problems/hallucinations	0.42	1.75	0.24	0.29	0.87	0.34	0.12	2.33	0.05
D5: Attention/memory	1.01	4.38	0.23	1.22	2.29	0.53	1.34	2.82	0.47
D6: Gastrointestinal	2.01	5.14	0.39	1.56	2.63	0.59	1.92	5.06	0.38
D7: Urinary	1.09	9.82	0.11	3.16	3.79	0.83	5.72	7.31	0.78
D8: Sexual function	1.02	5.32	0.19	2.20	3.91	0.56	3.78	8.57	0.44
D9: Miscellaneous	0.51	4.24	0.12	0.52	2.71	0.19	0.69	5.47	0.13

*NMSS* Non-Motor Symptoms Scale, *MSA* multiple system atrophy, *SD* standard deviation

## Discussion

In this prospective observational 2-year follow-up study, we investigated patients with early-stage MSA and observed significant progression of motor symptoms and NMS reflected by the UMSARS-I and II and the NMSS. Additionally, we observed differences between each item of the UMSARS, based on their sensitivity to change. The most sensitive to change items of UMSARS-I were those associated with motor functions, whereas items associated with autonomic dysfunction (orthostatic symptoms, urinary, sexual, and bowel function) showed lesser sensitivity to change over 1- and 2-year follow-ups. Items 3 (ocular motor dysfunction), 4 (tremor at rest), and 5 (action tremor) of UMSARS-II were less sensitive to change at the 1- and 2-year follow-ups.

A previous study reported an increase of 14 points in the median total UMSARS score at the 12-month follow-up and an increase of 22 points at the 24-month follow-up in patients with mild MSA (UMSARS-IV score ≤2) at baseline (*n* = 44) [14]. These results are consistent with those of our study; we observed a mean increase of 11.90 and 22.54 points at the 1- and 2-year follow-ups in our study. Despite the significant progression of the UMSARS, all items included in this scale did not show good ability to detect changes. We applied a SE (using the mean annual change divided by the SD of the change) to reflect the sensitivity of an item to change, as previously reported [9]. Studies from Western countries have shown that UMSARS-I items associated with motor functions, including speech, swallowing, handwriting, cutting food, hygiene, dressing, and walking were more sensitive to change; this finding concurs with that of our study [7, 9]. These items of the UMSARS-I also showed good ability to detect changes in Chinese patients with early-stage MSA. However, items associated with autonomic dysfunction (orthostatic symptoms, as well as urinary, sexual, and bowel function) were less sensitive to change in our study; these findings are supported by other studies of independent cohorts [7, 9]. Overall, items 9 (orthostatic symptoms), 10 (urinary function), and 12 (bowel function) of the UMSARS-I showed the poorest ability to detect change, which is attributable to the fact that severe autonomic failure is a fundamental clinical feature for diagnosis of MSA [7] or may be secondary to a floor effect in early-stage disease.

With regard to UMSARS-II, most items were sensitive to change, except for items 3 (ocular motor dysfunction), 4 (tremor at rest), and 5 (action tremor), which is consistent with the findings of previous studies [7, 9]. In a small-scale study of 70 patients with MSA, Palma et al. observed that tremor at rest and action tremor had limited ability to detect change [9], which is attributable to the fact that tremors (particularly, tremors at rest) are uncommon in MSA. Krismer et al. reported that in addition to items associated with tremors, oculomotor dysfunction was insensitive to change [7], which may at least partially be explained by an inaccurate description of specific oculomotor abnormalities because of difficulty in assessment.

Our study adds to the evidence that items 9 (orthostatic symptoms), 10 (urinary function), and 12 (bowel function) of the UMSARS-I and items 3 (ocular motor dysfunction), 4 (tremor at rest), and 5 (action tremor) of the UMSARS-II were less sensitive to change even during early-stage MSA. Based on data obtained from Western studies in combination with our findings, it is reasonable to conclude that the UMSARS may have a floor effect in early-stage and a ceiling effect in late-stage MSA. In view of the limitations of the UMSARS, optimization of UMSARS is warranted to improve its sensitivity to change.

This is the first study to use a comprehensive tool (the NMSS) to investigate the early progression of overall NMS in Chinese patients with early-stage MSA; we observed that NMS was prevalent as previously reported [11]. The frequency and severity of NMS significantly increased over the 2-year follow-up. A Spanish study, which used the NMSS to investigate changes in NMS over a 2-year follow-up, observed that only urinary and sexual dysfunction and sleep difficulties showed significant progression [14]. However, the study included only 80 patients with MSA, and only 42 patients successfully completed the 2-year follow-up. The findings of the aforementioned study are inconsistent with our findings; differences in sample size, disease duration at baseline, and ethnicity may have contributed to the discrepancies in results. As previously reported, the frequency and severity of fatigue in patients with MSA increased from baseline to the 1-year follow-up [13]. The results of the current study showed that the prevalence of sleep/fatigue increased from baseline to 1- and 2-year follow-ups, whereas the severity of sleep/fatigue increased from baseline until 2-year follow-up, which is attributable to the fact that we used different scales to evaluate fatigue.

The current study showed that most NMS increased significantly even during early-stage MSA. Accumulating evidence indicates that NMS is closely associated with poor quality of life in patients with MSA [11, 12]. Therefore, the management of NMS in MSA is important, and early intervention with regular monitoring is necessary throughout the course of the disease.

The NMSS and UMSARS have overlapping NMS items, including orthostatic, urinary, sexual, and bowel symptoms. Those items in the UMSARS and NMSS were less sensitive to change, particularly from baseline until the 1-year follow-up. Additionally, attention/memory, gastrointestinal, urinary, and sexual function domains of NMSS were more sensitive to change from baseline until the 2-year follow-up. NMSS contains more comprehensive NMS such as sleep/fatigue, mood/apathy, and attention/memory. Thus, we propose that NMSS can be used as a supplementary scale to evaluate various NMS of MSA in the clinical trials. Furthermore, the International Parkinson and Movement Disorder Society (MDS) task force is planning to revise the UMSARS and transform it into the MDS-UMSARS, which is a comprehensive scale covering the entire spectrum of MSA-specific symptoms, a patient-centered scale, and a set of virtually assessable items [18]. Taken together, the results of the current study may provide useful data to support the revision of the UMSARS, contributing to clinical trials in the future.

The following are the limitations of the current study: (a) The follow-up duration was relatively short. Long-term follow-up is difficult because MSA is a rare and rapidly progressive condition. (b) The single-center design of our study is likely to introduce systemic bias; multicenter studies are warranted in the future. However, we included patients from across more than five provinces of western China to reduce bias. (c) All patients were diagnosed clinically without postmortem confirmation.

## Conclusions

To our knowledge, this is the first study that reports significant progression of motor symptoms and NMS in a cohort of patients with early-stage MSA over a 2-year follow-up. This study highlights differences between individual items included in the UMSARS with regard to their sensitivity to change, even in patients with early-stage MSA. Optimization of UMSARS is warranted to improve its sensitivity to change. The results of the current study may provide useful data to support the revision of the UMSARS.

## Supplementary Information

**Additional file 1: Table S1.** Baseline demographics of MSA. **Table S2.** The comparison of frequency of each NMSS domain at baseline and 1- and 2-year follow-up.

## Data Availability

The datasets used and/or analyzed during the current study are available from the corresponding author on reasonable request.

## Abbreviations

LEDD: Levodopa equivalent daily dose
MDS: International Parkinson and Movement Disorder Society
MSA: Multiple system atrophy
MSA-C: MSA with predominant features of cerebellar ataxia
MSA-P: MSA with predominantly parkinsonian features
NMS: Non-motor symptoms
NMSS: Non-Motor Symptoms Scale
SCA: Spinocerebellar ataxia
SD: Standard deviation
SE: Standardized effect
UMSARS: Unified Multiple System Atrophy Rating Scale
