# Supplementary material for: Longitudinal evolution of motor and non-motor symptoms in early-stage multiple system atrophy: a 2-year prospective cohort study
Source: BMC Med. 2022 Nov 17;20:446. doi: 10.1186/s12916-022-02645-1 (PMC9670051; doi:10.1186/s12916-022-02645-1)
Supplement: Supplementary file 1 — Additional file 1: Table S1. Baseline demographics of MSA. Table S2. The comparison of frequency of each NMSS domain at baseline and 1- and 2-year follow-up. [file 12916_2022_2645_MOESM1_ESM.docx]

Table S1 Baseline demographics of MSA.

| Variables | MSA | | | |
| --- | --- | --- | --- | --- |
|  | total (n=246) | Non-loss of FU(n=97) | Loss of FU(n=149) | p value |
| Age | 59.41±7.68 | 58.45±8.02 | 60.04±7.41 | 0.113 |
| Sex, n (%) |  |  |  | 0.114 |
| Male | 132(53.7） | 46(47.4) | 86(57.7) |  |
| Female | 114(46.3) | 51(52.6) | 63(42.3) |  |
| Age of onset | 57.69±7.65 | 56.38±7.38 | 58.38±7.38 | 0.078 |
| Disease duration | 1.72±0.76 | 1.83±0.78 | 1.66±0.75 | 0.092 |
| Diagnostic subtype, n (%) | |  |  | 0.826 |
| MSA-P | 112(45.5) | 45(46.4) | 67(45.0) |  |
| MSA-C | 134(54.5) | 52(53.6) | 82(55.0) |  |
| UMSARS-I | 13.47±5.50 | 12.90±5.27 | 13.84±5.63 | 0.190 |
| UMSARS-II | 15.56±5.93 | 14.74±5.69 | 16.09±6.04 | 0.082 |
| OH, n (%) | 90(36.6) | 41(42.3) | 49(32.9) | 0.135 |
| UMSARS-IV | 1.78±0.77 | 1.67±0.77 | 1.85±0.77 | 0.082 |
| UMSARS-total score | 29.02±10.52 | 27.64±10.16 | 29.93±10.68 | 0.096 |
| LEDD | 156.56±230.04 | 96.52±174.28 | 195.64±252.98 | 0.001* |
| NMSS |  |  |  |  |
| D1: Cardiovascular | 3.07±4.00 | 2.80±3.87 | 3.25±4.08 | 0.395 |
| D2: Sleep/fatigue | 6.82±6.93 | 6.69±7.48 | 6.90±6.57 | 0.818 |
| D3: Mood/apathy | 10.87±14.93 | 9.82±13.66 | 11.54±15.71 | 0.379 |
| D4: Perceptual problems/hallucinations | 0.15±0.88 | 0.10±0.34 | 0.19±1.10 | 0.462 |
| D5: Attention/memory | 3.43±4.28 | 3.26±4.05 | 3.54±4.43 | 0.618 |
| D6: Gastrointestinal | 4.10±4.55 | 4.10±4.59 | 4.09±4.54 | 0.988 |
| D7: Urinary | 11.95±11.13 | 11.81±11.58 | 12.03±10.88 | 0.880 |
| D8: Sexual function | 4.82±7.24 | 3.98±6.20 | 5.37±7.81 | 0.141 |
| D9: Miscellaneous | 4.22±4.86 | 3.88±4.90 | 4.44±4.83 | 0.378 |
| NMSS total score | 49.42±34.55 | 46.45±32.97 | 51.35±35.52 | 0.278 |

MSA: multiple system atrophy; MSA-P: multiple system atrophy with predominant parkinsonism; MSA-C: multiple system atrophy with predominant cerebellar ataxia; LEDD: levodopa equivalent daily dose; UMSARS: Unified Multiple System Atrophy Rating Scale; OH: orthostatic hypotension; FU: follow-up.

* Significant difference.

Table S2 The comparison of frequency of each NMSS domain at baseline and 1- and 2-year follow-up

| NMSS | Baseline (n=246) | 1-year follow-up (n=246) | 2-year follow-up (n=97) | p value^#^ | Post hoc tests^#^ |
| --- | --- | --- | --- | --- | --- |
| D1: Cardiovascular | 155 (63.0%) | 192 (78.05%) | 85 (87.6%) | <0.001* | **a,b,c** |
| D2: Sleep/fatigue | 189 (76.8%) | 225 (91.5%) | 93 (95.9%) | <0.001* | **a,b** |
| D3: Mood/apathy | 173 (70.3%) | 212 (86.2%) | 93 (95.9%) | <0.001* | **a,b,c** |
| D4: Perceptual problems/hallucinations | 20 (8.1%) | 50 (20.3%) | 21 (21.6%) | <0.001* | **a,b** |
| D5: Attention/memory | 168 (68.3%) | 215 (87.4%) | 87 (89.7%) | <0.001* | **a,b** |
| D6: Gastrointestinal | 179 (72.8%) | 229 (93.1%) | 96 (99.0%) | <0.001* | **a,b** |
| D7: Urinary | 197 (80.1%) | 228 (92.7%) | 96 (99.0%) | <0.001* | **a,b** |
| D8: Sexual function | 106 (43.1%) | 133 (54.1%) | 63 (64.9%) | <0.001* | **a,b,c** |
| D9: Miscellaneous | 162 (65.9%) | 204 (82.9%) | 89 (91.8%) | <0.001* | **a,b,c** |

NMSS: non-motor symptoms scale.

^#^ Adjusted by baseline age and sex.

* Significant difference.

Post hoc tests (Bonferroni correction):

Baseline vs 1-year follow-up: **a**, significant;

Baseline vs 2-year follow-up: **b**, significant;

1- vs 2-year follow-up: **c**, significant.
